# Systemic triplet therapy for metastatic hormone-sensitive prostate cancer: A systematic review and network meta-analysis

**DOI:** 10.3389/fphar.2022.955925

**Published:** 2022-10-06

**Authors:** Tengteng Jian, Yang Zhan, Kebang Hu, Liang He, Sunmeng Chen, Rui Hu, Ji Lu

**Affiliations:** ^1^ Department of Urology, The First Hospital of Jilin University, Changchun, China; ^2^ School of Life Sciences, Jilin University, Changchun, China

**Keywords:** metastatic hormone-sensitive prostate cancer, Docetaxel, systemic therapy, triplet therapy, network meta-analysis

## Abstract

**Purpose:** To perform a systematic review and network meta-analysis to compare the efficacy and safety of currently available docetaxel-based systemic triplet therapies for metastatic hormone-sensitive prostate cancer (mHSPC).

**Methods:** We searched for eligible publications in PubMed, Embase, and Cochrane CENTRAL. Improvements in overall survival (OS) and radiographic progression-free time (rPFS) were compared indirectly using network meta-analysis and evaluated using the surface under the cumulative ranking curve (SUCRA). Other secondary endpoints, such as time to castration-resistant prostate cancer and/or adverse events (AEs), were also compared and evaluated.

**Results:** Five trials were selected and analyzed using a network meta-analysis. Compared to androgen deprivation therapy (ADT) plus docetaxel, darolutamide (hazard ratio [HR]: 0.68, 95% credible interval [CrI]: 0.57–0.80) and abiraterone (HR: 0.75, 95% CrI: 0.59–0.95) triplet therapy had significantly longer OS, and darolutamide triplet therapy was the first treatment ranked. Abiraterone (HR: 0.49, 95% CrI: 0.39–0.61) and enzalutamide (HR: 0.52, 95% CrI: 0.30–0.89) had significantly better rPFS than ADT plus docetaxel; however, all three therapies, including abiraterone, apalutamide, and enzalutamide, were the best options with a similar SUCRA. At most secondary endpoints, systemic triplet therapy was superior to ADT plus docetaxel. The risk of any AEs in darolutamide or abiraterone triplet therapy was comparable with ADT plus docetaxel (odds ratio [OR]: 2.53, 95% credible interval [CrI]: 0.68–12.63; OR: 1.07, 95% CrI: 0.03–36.25). Abiraterone triplet therapy had an increased risk of grade≥3 AEs (OR: 1.56, 95% CrI: 1.15–2.11).

**Conclusion:** Systemic triplet therapy was more effective than ADT plus docetaxel for mHSPC. Of the triplet therapy regimens, darolutamide ranked first in terms of improved OS. Abiraterone and enzalutamide triplet ranked first in terms of rFPS, however, it did not confer a statistically difference among all triplet regimens. The overall risk of AEs was comparable. More studies are required for current and potential combinations of systemic triplet therapy.

## 1 Introduction

Prostate cancer (PCa) is the second most commonly diagnosed malignancy in men worldwide, with about 1.4 million newly diagnosed cases and 375,304 deaths in 2020 (Sung et al., 2021). The 5-year relative survival rate for PCa with all stages combined is 98%. However, the 5-year survival rate for patients with metastatic prostate cancer is only about 30% (Siegel et al., 2022). Androgen deprivation therapy (ADT) is the mainstay of therapy for metastatic hormone-sensitive prostate cancer (mHSPC). To obtain better efficacy, ADT-based combined therapies have been tried. The earliest combination therapy was ADT combined with first-generation nonsteroidal antiandrogen, often referred to as maximal or complete androgen blockade. But the benefit of this therapy was only 2–3% of survival advantage compared with ADT monotherapy (Prostate Cancer Trialists' Collaborative Group, 2000).

In recent years, several other combined systemic therapies have been introduced for mHSPC, including ADT combined with docetaxel, abiraterone, apalutamide, and enzalutamide. Among these therapies, the underlying antitumor mechanism of docetaxel is the cytotoxic effects caused by microtubule stabilization, mitotic arrest, and apoptotic cell death. The mechanisms of the new androgen receptor-targeted agent (ARTA) include inhibition of androgen synthesis *via* CYP17 enzymes (abiraterone), blocking androgen-induced AR activation and nuclear translocation, and inhibiting DNA binding and AR-mediated transcription (apalutamide and enzalutamide) (Crawford et al., 2018). Since the addition of these agents to ADT have shown survival benefits than ADT alone, these combined therapies have been the standard treatments for mHSPC currently.

Due to the distinct and complementary antitumor mechanisms of docetaxel and ARTAs, it is natural to wonder whether the combination of these two with ADT for mHSPC would produce better therapeutic effects. However, few studies that included ADT plus two active agents, i.e., the systemic triplet therapy, have been conducted to treat mHSPC. Recently, it was reported that ADT plus docetaxel combined with abiraterone or darolutamide resulted in better efficacy than ADT plus docetaxel (Fizazi et al., 2022; Smith et al., 2022). However, it is unclear whether other triplet therapy combinations exist and which one is better. Several systematic reviews and network meta-analyses (NMAs) have been conducted to summarize and compare systemic doublet therapies (ADT plus one agent) for mHSPC (Rydzewska et al., 2017; Vale et al., 2018; Wang et al., 2021; Mori et al., 2022). However, until now, few systematic review and meta-analyses for systemic triplet therapy published, and there has been no indirect comparison of the efficacy and safety of individual systemic triplet therapy for mHSPC. Therefore, we performed a systematic review of all docetaxel-based triplet therapies and indirectly compared the efficacy and safety of these therapies through NMA.

## 2 Methods

### 2.1 Study design

The study protocol was registered in the International Prospective Register of Systematic Reviews database (PROSPERO: CRD42022324980). This study followed the updated Preferred Reporting Items for Systematic Reviews and Meta-analyses (PRISMA) reporting guideline and its extension for network meta-analysis (Hutton et al., 2015; Page et al., 2021).

### 2.2 Literature search

We searched PubMed, Embase, and Cochrane CENTRAL databases to identify reports published before April 2022 on systemic therapy for mHSPC. Trials included mHSPC treatment and docetaxel-based systemic therapy. Some subjects and free words related to prostate cancer, metastasis, docetaxel, and randomized trials were used. In addition, we reviewed clinical trial registries and relevant abstracts presented at major conferences, including the American Society of Clinical Oncology and the European Society for Medical Oncology. A detailed database search strategy is presented in Supplementary Table S1.

### 2.3 Inclusion and exclusion criteria

A trial was included in the systematic review if: 1) it includes patients in the group or subgroup that should be treated with systemic therapy containing ADT plus docetaxel with or without another agent; 2) the trial reported the efficacy of overall survival (OS) or progression-free survival (PFS). Trials were excluded if there were: 1) castration-resistant prostate cancer; 2) patients without metastatic disease; and 3) observational studies, reviews, cohorts, replies from authors, and case reports. Initial screening was performed to identify ineligible reports based on the title and abstract of the article. They indicated the reasons for their exclusion. Potentially relevant reports were reviewed in full text, and their relevance was confirmed after data extraction. The title and abstract screening and full text screening were performed independently by two investigators (TJ and YZ). Disagreements were resolved by consensus among the co-authors.

### 2.4 Data collection

Two investigators (KH and LH) independently extracted the following information from the included articles: the first author’s name, year of publication, trial name, number of patients, patient age, performance status, inclusion criteria, agents, treatment dose, treatment duration, disease volume, and outcome definition. Furthermore, hazard ratios (HRs) and 95% confidence intervals associated with the primary endpoint (OS and PFS), secondary endpoints (time to castration resistance, time to PSA progression, time to first symptomatic skeletal event, time to new antineoplastic therapy), and adverse events (AE) were also collected. All discrepancies regarding data extraction were subjected to a consensus among the co-authors.

### 2.5 Quality evaluation

The risk of bias for each trial was assessed according to the Cochrane Collaboration tool for assessing the risk of bias (Higgins et al., 2011). This tool assesses selection bias (random sequence generation and allocation concealment), performance bias, detection bias, attrition bias, reporting bias, and bias from other sources. The certainty or quality of evidence was assessed with Grading of Recommendations Assessment, Development and Evaluation (GRADE) tool (Atkins et al., 2004; Guyatt et al., 2011). This tool categorized the quality of evidence into four levels (high, moderate, low, and very low). The certainty of evidence began as high, which could be downrated to moderate, low, or very low according to five domains (risk of bias, inconsistency, imprecision, indirectness, and publication bias). The risk of bias and certainty of evidence were evaluated independently by two authors (SC and RH).

For a network meta-analysis to be valid, three assumptions should be met, including homogeneity, consistency, and transitivity (Salanti, 2012). Homogeneity assumption refers to that among available trials the direct comparisons should be sufficiently homogeneous for each intervention group. Consistency assumption refers to that effect estimates derived from direct head-to-head comparisons and indirect comparisons should be consistent. It is required if a treatment network contains a closed loop of interventions. Transitivity assumption refers to that included trials should be clinically and methodologically sufficiently comparable.

### 2.6 Statistical analyses

Network plots were used to illustrate the connectivity of the treatment network in terms of the OS, rPFS, and other secondary endpoints. For indirect comparison, a Bayesian network meta-analysis was conducted using fixed- and random-effects models. We reported the results from the fixed-effects models to account for inter-study heterogeneity and all comparisons examined in only one trial. Relative treatment effects were expressed as HRs and 95% credible intervals (CrIs) (Woods et al., 2010; van Valkenhoef et al., 2012). The ranking probability of treatments for each outcome was estimated using the surface under the cumulative ranking curve (SUCRA) (Salanti et al., 2011). Comparisons between the two therapy groups are shown in league tables. For AEs in selected trials, comparisons between groups or subgroups were performed to estimate the odds ratio (ORs) and 95% CrI (van Valkenhoef et al., 2012). All statistical analyses were performed using R version 4.1.2 (R Foundation for Statistical Computing, Vienna, Austria); the statistical difference was set *p* < 0.05.

## 3 Results

### 3.1 Study selection and characteristics

**FIGURE 1:**
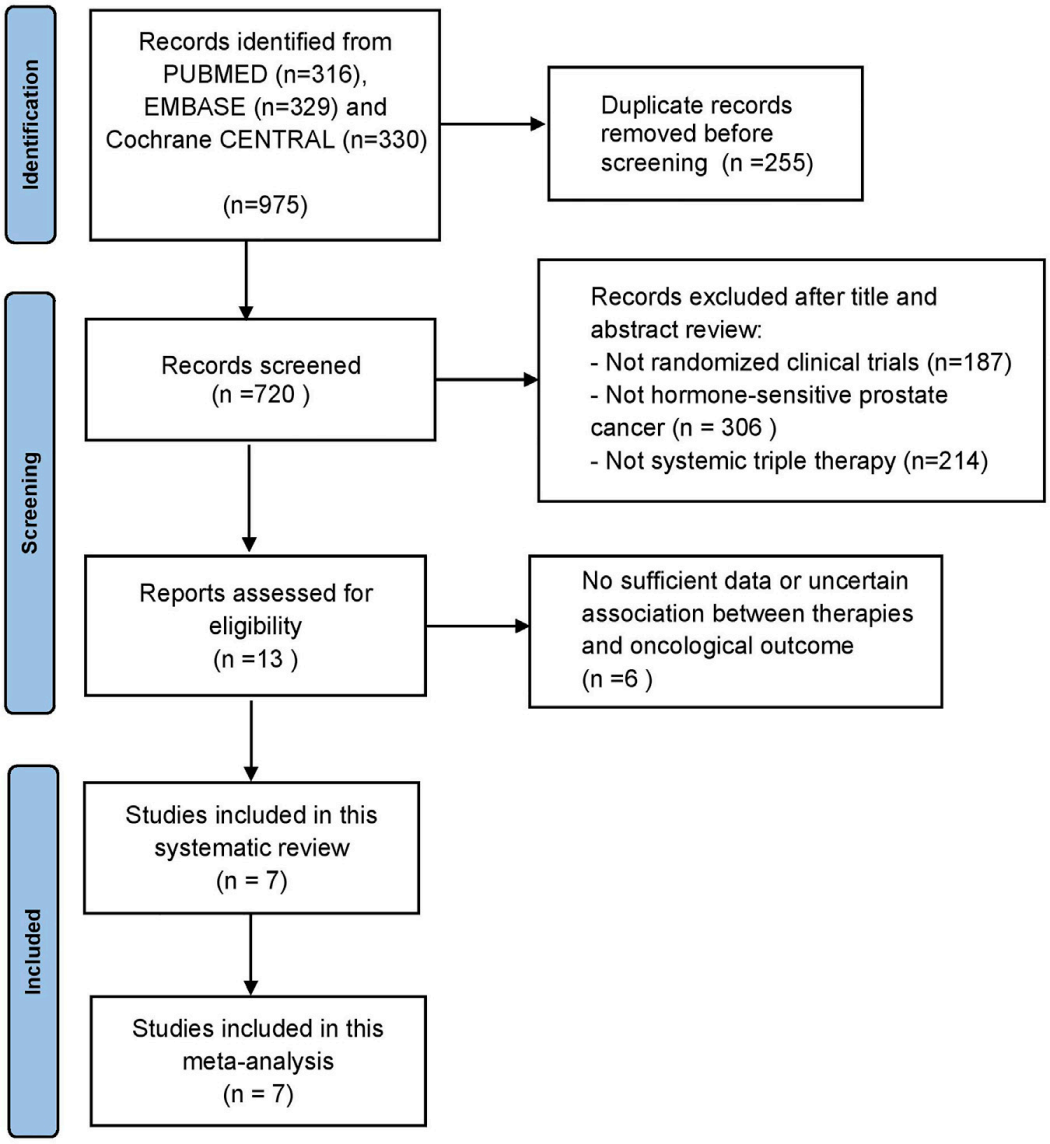
The PRISMA flow chart detailing the study selection process.

**FIGURE 2:**
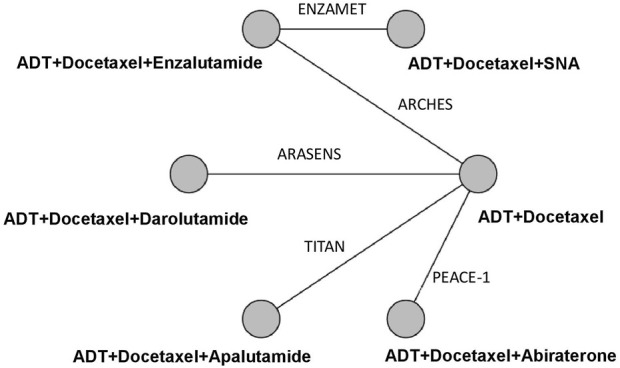
Network Graph of Trials Comparison. The nodes (circles) represent comparative therapy, and the edges (lines) show which therapies have been compared. The labels on the edges represent the names of RCTs comparing therapies. SNA: Standard nonsteroidal antiandrogen.

Our initial search identified 975 publications; however, 720 publications were retained after eliminating duplicates. After title and abstract screening, 707 articles were excluded, and full-text reviews were performed for 13 articles (Figure 1). Based on the selection criteria, we identified seven studies reporting five randomized controlled trials (RCTs) comprising 5 804 patients in total (Armstrong et al., 2019; Chi et al., 2019; Davis et al., 2019; Azad et al., 2021; Chi et al., 2021; Fizazi et al., 2022; Smith et al., 2022). After selecting eligible subgroups in some trials, a total of 2 836 patients were included in the systematic review and NMA. The characteristics and data extracted from the seven studies are summarized in Table 1. These studies were published between 2019 and 2022, and six therapies were compared. A total of 1 415 patients were treated with ADT plus docetaxel combined with control or standard nonsteroidal anti-androgen treatments, and 1 421 patients were treated with systemic triplet therapy, ADT plus docetaxel combined with one of the following ARTA, including abiraterone, enzalutamide, apalutamide, or darolutamide. A network graph of treatment comparisons is presented in Figure 2.

### 3.2 Quality evaluation

The risk of bias assessment results are presented in Supplementary Figure S1. All included studies were randomized prospective clinical trials. ENZAMET and PEACE-1 are open trials, and the studies were considered to be at potentially high risk for blinding of participants and personnel. Two of the five trials were unclear risk for random sequence generation, and three of the five trials were unclear risk for other bias. Detailed assessment criteria for each item in all included trials were listed in Supplementary Table S4.

The certainty of evidence for time to first symptomatic skeletal event and time to new antineoplastic therapy were high, while adverse events (any grade) had low certainty of evidence due to study limitation and imprecision. Other outcomes had moderate certainty of evidence due to study limitation. Detailed interpretation for grades of evidence was listed in Supplementary Table S5.

In our study, funnel plot showed that these was no significant publication bias in the pooled analyses (Supplementary Figure S2). In NMA, for each intervention comparison there is only one trial available, thus the study meets the homogeneity assumption. Since there was no comparison between direct and indirect evidence, and no closed loop existed in our NMA, local inconsistency analysis was not performed. Thus, the consistency assumption was met. According to the inclusion and exclusion criteria, and the data summarized in Table 1, the transitivity assumption was met in clinical and methodological aspects across the included trials.

**TABLE 1:** Characteristics of Clinical Trials Included in the Network Meta-Analyses.

Trials	Overall pateints	Patients with DOC	Experimental arm (*n*)	Control arm (*n*)	Median age (range) (E vs C)	ECOG PS score,n(%) (E vs C)	Gleason score at initial diagnosis,n(%) (E vs C)	Metastatic volume (E vs C)	Median PSA(range), ng/ml (E vs C)	DOC initiation	Cycles of DOC	Efficacy outcomes in NMA^c^
ARCHES (Armstrong et al., 2019; Azad et al., 2021)	1150	205	ADT+Docetaxel+Enzalutamide (103)	ADT+Docetaxel +Placebo (102)	67(46–84) vs. 68(42–83)	0: 76(73.8) vs. 76(74.5) 1: 26(25.2) vs. 26(25.5) Missing:1(1)	≤7: 23(22.3) vs. 26(25.5) 8-10: 76(73.8) vs. 72(70.6) Missing: 8(3.9)	HV: 73(70.9) vs. 72(70.6) LV: 30(29.1) vs. 30(29.4)	0.8(0.0-493.7) vs. 0.76(0.0-280.8)	Prior	Full 6 cycles used in 86% of patients	OS, rPFS, time to PSA progression, time to CR, time to first SSE, time to new antineoplastic therapy
ENZAMET^a^ (Davis et al., 2019)	1125	503	ADT+Docetaxel +Enzalutamide (254)	ADT+Docetaxel +SNA (249)	69.2(IQR:63.2–74.5) vs. 69.0 (IQR63.6–74.5)	0: 405(72) vs. 405(72) 1-2: 158(28) vs. 157(28)	≤7: 152(27) vs. 163(29) 8-10: 353(60) vs. 321(57) Missing: 76(13) vs. 78(14)	HV: 177(69.7) vs. 179(71.9) LV: 77(30.3) vs. 70(28.1)	NR	Prior (35%) and concomitant (65%)	Full 6 cycles used in 71% of patients	OS, PSA progression-free survival, Clinical Progression-Free Survival
TITAN^a^ (Chi et al., 2019; Chi et al., 2021)	1052	113	ADT+Docetaxel+Apalutamide (58)	ADT+Docetaxel+Placebo (55)	69 (45–94) vs. 68 (43–90)	0: 328(62.5) vs. 348(66.0) 1-2: 197(37.5) vs. 179(34)	≤7: 174(33.1) vs. 169(32.1) 8-10: 351(66.9) vs. 358(67.9)	HV: 325(61.9) vs. 335(63.6) LV: 200(38.1) vs. 192(36.4)	5.97(0-2682) vs 4.02(0-2229)	Prior	In median, 6 cycles used	OS, rPFS
PEACE-1 (Fizazi et al., 2022)	1172	710	ADT+Docetaxel+Abiraterone+RT(+/-) (355)	ADT+Docetaxel +RT(+/-) (355)	66(IQR:60-70) vs. 66(IQR:59-70)	0: 250(70%) vs. 246(69%) 1-2: 105(30%) vs. 109(31%)	≤7: 79(23%) vs. 71(20%) 8-10: 270(77%) vs. 276(80%) Missing: 6(2%) vs. 8(2%)	HV: 224(63) vs. 232(65) LV: 131(37) vs. 123(35)	14(2-59) vs 12(3-60)	Concomitant	Full 6 cycles used in 100% of patients	OS, rPFS, CRPC-free survival
ARASENS^b^ (Smith et al., 2022)	1305	1305	ADT+Docetaxel+Darolutamide (651)	ADT+Docetaxel +Placebo (654)	67(41–89) vs. 67 (42–86)	0: 466(71.6) vs. 462 (70.6) 1: 185(28.4) vs. 190(29.1) Missing: 2(1)	≤7: 122(18.7) vs. 118(18.0) 8-10: 505(77.6) vs. 516(78.9) Missing: 24(3.7) vs. 20(3.1)	M1a: 23(3.5) vs. 16(2.4) M1b: 517(79.4) vs. 520(79.5) M1c: 111(17.1) vs. 118(18.0)	30.3(0.0-9219.0) vs. 24.2(0.0-11947.0	Concomitant	Full 6 cycles used in 100% of patients	OS, time to CRPC, time to first SSE, time to initiation of subsequent systemic antineoplastic therapy

a The characteristics of ENZAMET and TITAN were from overall population. Metastatic Volume in ENZAMET was from DOC population.

b No high or low volume of metastasis is reported in ARASENS.

c Definitions and results of efficacy outcomes in NMA are listed in Supplementary Tables S2, S3.

Abbreviations: ADT, androgen-deprivation therapy; ARASENS, ODM-201 in Addition to Standard ADT and Docetaxel in Metastatic Castration Sensitive Prostate Cancer; ARCHES, A Study of Enzalutamide Plus Androgen Deprivation Therapy (ADT) Versus Placebo Plus ADT in Patients With Metastatic Hormone Sensitive Prostate Cancer (mHSPC); C, Control Arm; DOC, docetaxel; ECOG PS, Eastern Cooperative Oncology Group Performance Status; E, Experimental Arm ; ENZAMET, Enzalutamide in First Line Androgen Deprivation Therapy for Metastatic Prostate cancer; HL, high volume; LV, low volume; NR, not reported; OS, overall survival; PEACE-1, A Phase IIIStudy for Patients With Metastatic Hormone-natïve Prostate Cancer; rPFS, Radiographic progression-free survival; RT, radiotherapy; SNA, standard nonsteroidal antiandrogen (bicalutamide, nilutamide or flutamide); TITAN, Targeted Investigational Treatment Analysis of Novel Anti-androgen.

### 3.3 Overall survival

**FIGURE 3:**
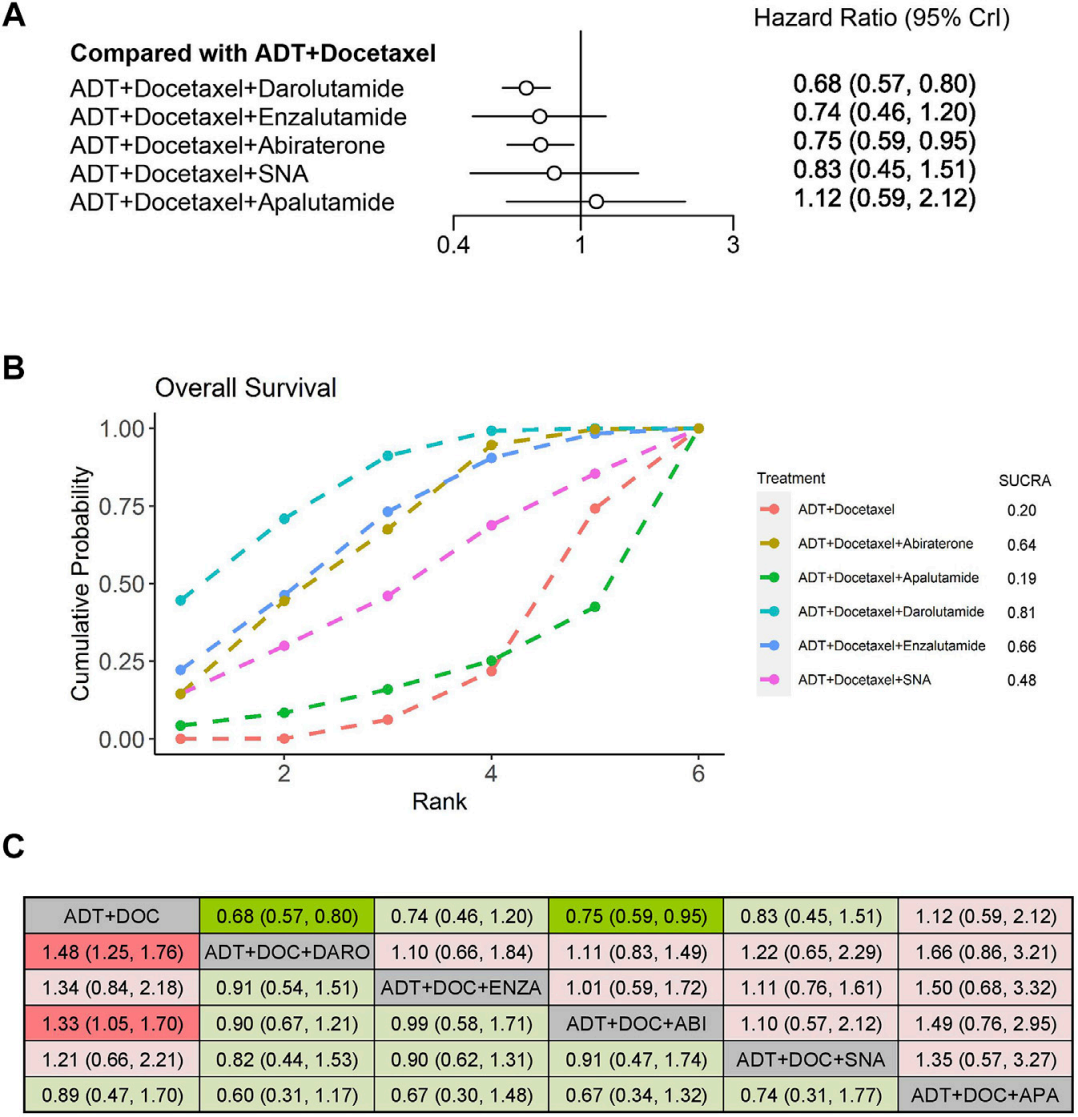
Comparison of systemic triplet therapy for improving OS. **(A)** Forest plot representing HR for each therapy compared with ADT plus docetaxel. HR and 95% CrI are represented. **(B)** SUCRA plot showing the treatment ranking of therapies. **(C)** League table of network meta-analysis comparing the OS effects of systemic therapies. Comparison is located at the intersection of the column-defining treatment and the row-defining treatment. The results are presented in HR with 95% CrI. HR>1 (red color) favors row-defining treatment, and HR<1 (green color) favors column-defining treatment. Dark red or green color represents the results with statistical significance. ADT, androgen deprivation therapy; SNA, Standard nonsteroidal antiandrogen; DO,: Docetaxel; ABI, Abiraterone, ENZA, Enzalutamide; DARO, Darolutamide; APA, Apalutamide.

We evaluated the OS improvement of four systemic triplet therapies for mHSPC using an NMA. Compared with ADT plus docetaxel, the triplet therapy of ADT plus docetaxel with darolutamide or abiraterone showed significantly improved OS (HR: 0.68, 95% CrI: 0.57–0.80; HR: 0.75, 95% CrI: 0.59–0.95), with a reduction in the risk of death by 32% and 25%, respectively (Figure 3A). According to the treatment ranking analysis, darolutamide triplet therapy had the highest probability of providing maximum OS, with a SUCRA of 0.81, followed by enzalutamide and abiraterone (Figure 3B). However, among the five triplet therapies, no significant difference was observed in OS improvement between any two groups (Figure 3C).

### 3.4 Radiographic progression-free time

**FIGURE 4:**
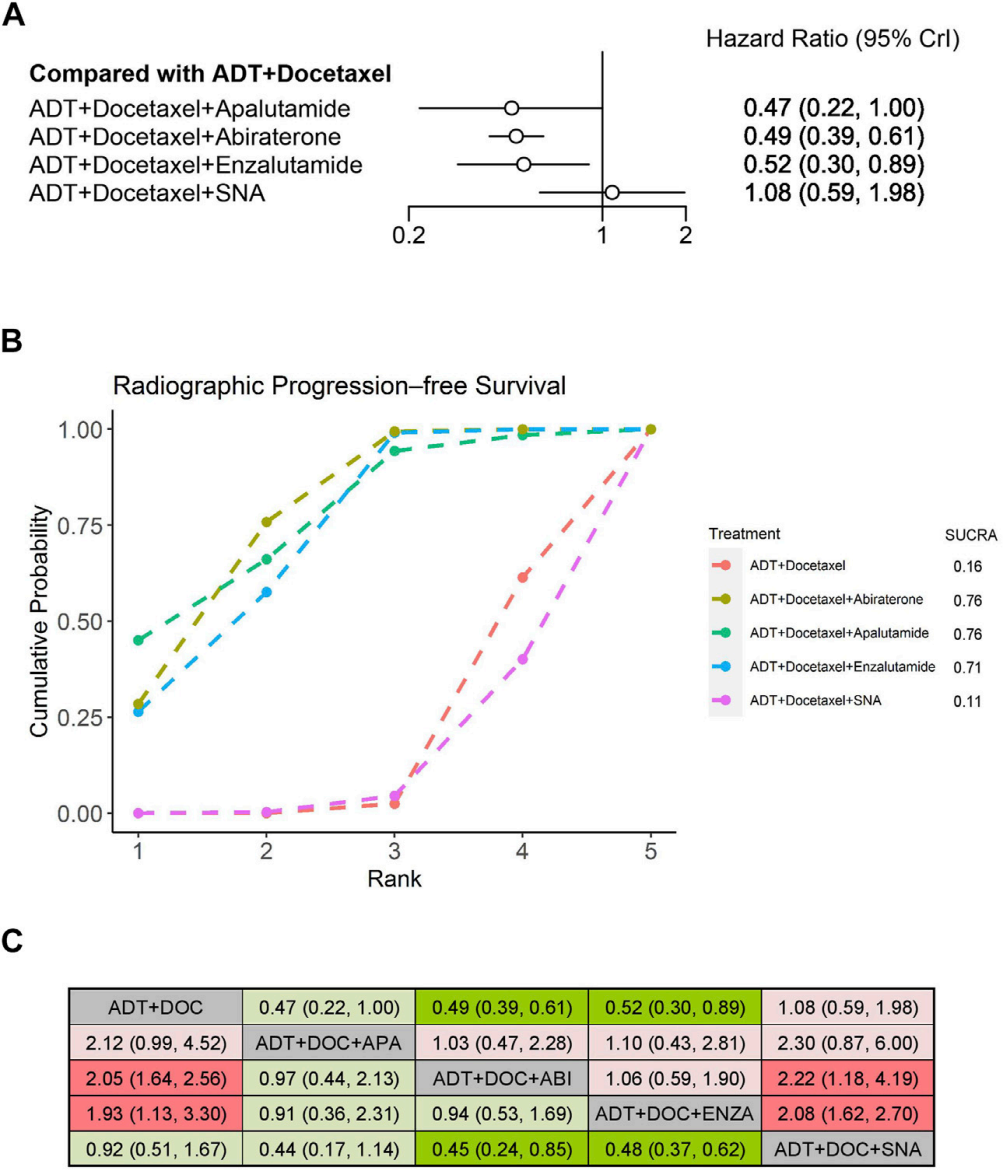
Comparison of systemic triplet therapy for improving rPFS. **(A)** Forest plot representing HR for each therapy compared with ADT plus docetaxel. HR and 95% CrI are represented. **(B)** SUCRA plot showing the treatment ranking of therapies. **(C)** League table of network meta-analysis comparing the rPFS effects of systemic therapies. Comparison is located at the intersection of the column-defining treatment and the row-defining treatment. The results are presented in HR with 95% CrI. HR>1 (red color) favors row-defining treatment, and HR<1 (green color) favors column-defining treatment. Dark red or green color represents the results with statistical significance. ADT, androgen deprivation therapy; SNA, Standard nonsteroidal antiandrogen; DOC, Docetaxel; ABI, Abiraterone; ENZA, Enzalutamide; APA, Apalutamide.

We evaluated improvement in rPFS for mHSPC among the three systemic therapies using NMA. Compared with ADT plus docetaxel alone, treatments that prolonged rPFS significantly when combined with ADT plus docetaxel included abiraterone (HR: 0.49, 95% CrI: 0.39–0.61) and enzalutamide (HR: 0.52, 95% CrI: 0.30–0.89) (Figure 4A). According to the treatment ranking analysis, abiraterone, apalutamide, and enzalutamide were the first three additions to triplet therapies that were most likely to achieve the best treatment, with similar probabilities of 76%, 76%, and 71%, respectively (Figure 4B). ADT plus docetaxel combined with a standard nonsteroidal antiandrogen (SNA), which was not considered as the triplet therapy, did not improve rPFS. It was significantly inferior to abiraterone or enzalutamide triplet therapy but comparable with apalutamide triplet therapy or ADT plus docetaxel doublet therapy when an indirect comparison between both therapies was performed (Figure 4C).

### 3.5 Secondary endpoints

**FIGURE 5:**
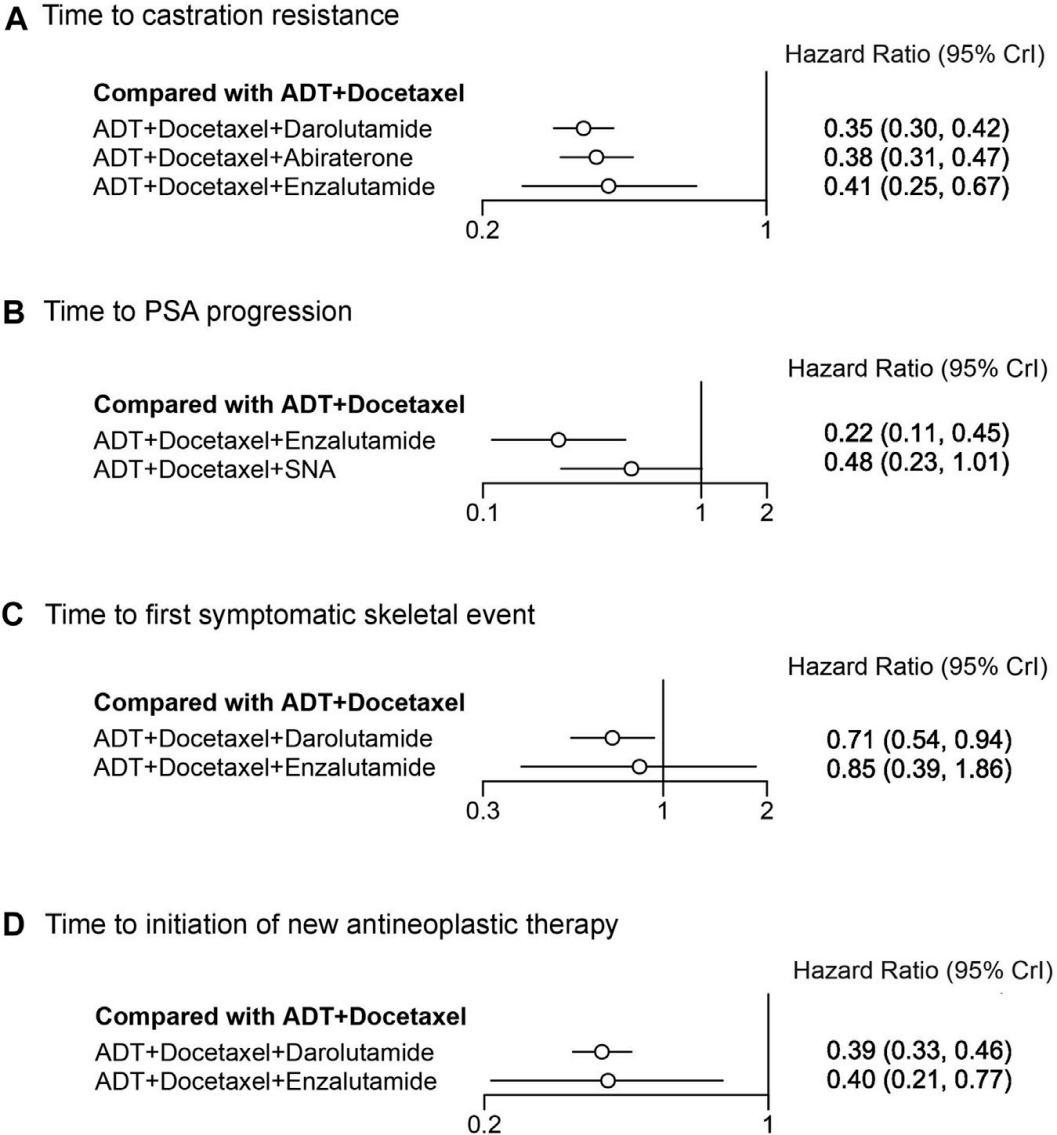
Forest plot representing a comparison of systemic triplet therapy for improving secondary endpoints compared with ADT plus docetaxel. HR and 95% CrI are represented. **(A)** Time to castration resistance; **(B)** time to PSA progression; **(C)** time to the first symptomatic skeletal event; **(D)** time to initiation of new antineoplastic therapy.

Some secondary endpoints in the triplet trials were analyzed and indirectly compared using NMA (Figure 5). The time to castration resistance was significantly extended in patients receiving darolutamide, abiraterone, and enzalutamide triplet therapy, with HR of 0.35 (95% CrI: 0.30–0.42), 0.38 (95% CrI: 0.31–0.47), and 0.41 (95% CrI: 0.25–0.67), respectively. For other secondary endpoints, enzalutamide triplet therapy also prolonged the time to PSA progression (HR: 0.22, 95% CrI: 0.11–0.45) and the time to initiation of new antineoplastic therapy (HR: 0.40, 95% CrI: 0.21–0.77). In addition to the time to initiation of new antineoplastic therapy, darolutamide triplet therapy also extended the time to the first symptomatic skeletal event (SSE) (HR 0.71, 95% CrI: 0.54–0.94), which, however, was not improved by enzalutamide. In the treatment ranking analysis, darolutamide triplet therapy ranked first in the time of castration resistance, first SSE, and initiation of new antineoplastic therapy, compared with other therapies (Supplementary Figure S3). Other comparisons between therapies for secondary endpoints are presented in league tables (Supplementary Figure S4).

### 3.6 Adverse events

**FIGURE 6:**
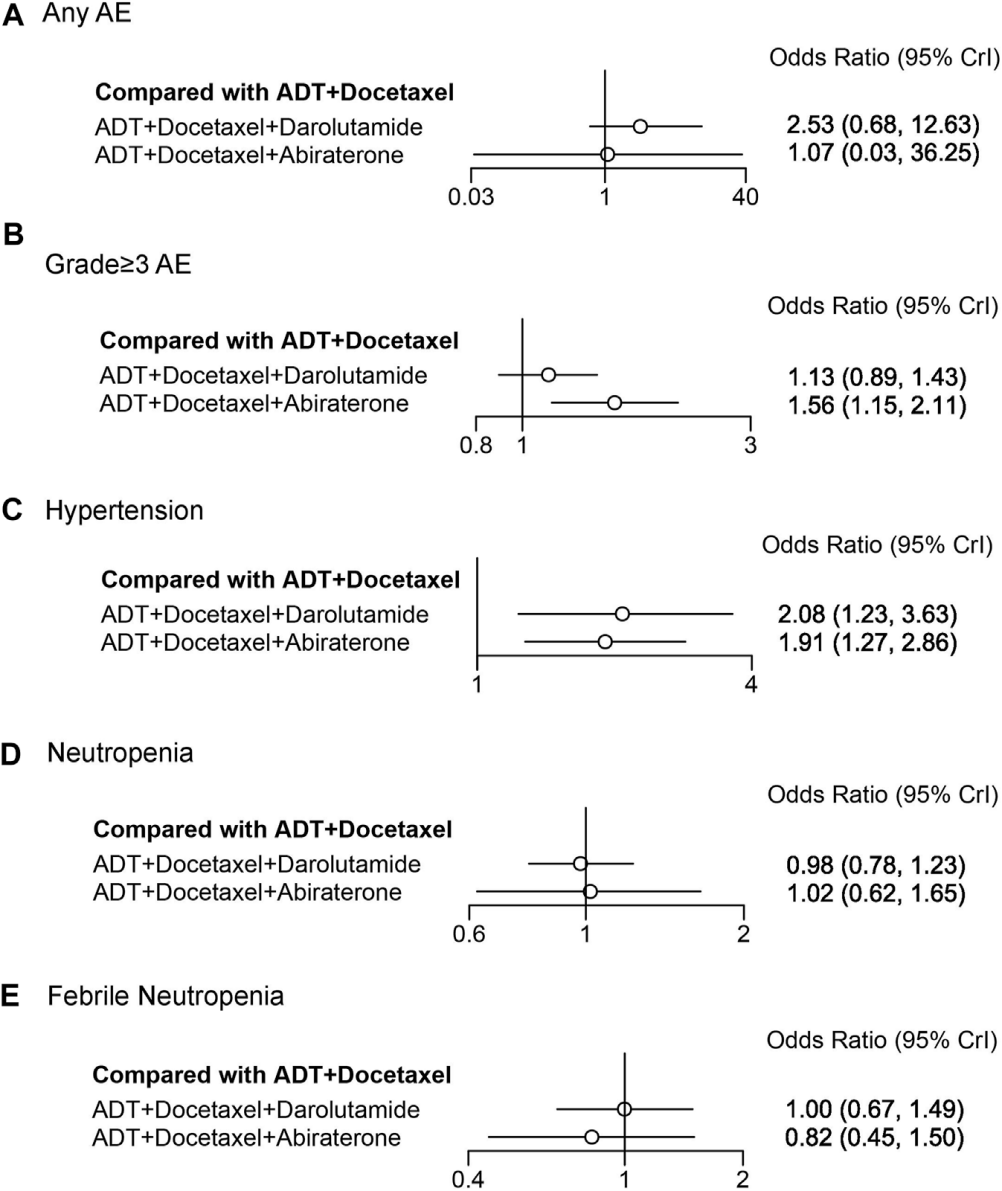
Forest plot representing a comparison of adverse events for systemic triplet therapy compared with ADT plus docetaxel. HR and 95% CrI are represented. **(A)** Any AE; **(B)** grade≥3AE; **(C)** hypertension; **(D)** neutropenia; **(E)** febrile neutropenia.

We assessed adverse events between darolutamide and abiraterone triplet therapies using NMA (Figure 6). Neither of these therapies demonstrated an increased risk of any AEs compared with ADT plus docetaxel (OR 2.53, 95% CrI: 0.68–12.63; OR 1.07, 95% CrI: 0.03–36.25). However, an increased risk of grade ≥3 AEs was identified in abiraterone triplet therapy (OR 1.56, 95% CrI: 1.15–2.11). Three AEs with a high incidence were selected for further comparison. The risk of hypertension in both therapies was significantly increased (OR 2.08, 95% CrI: 1.23–3.63; OR 1.91, 95% CrI: 1.27–2.86). Neither darolutamide nor abiraterone triplet therapy was associated with an increased risk of neutropenia or febrile neutropenia. Other detailed AEs and their corresponding odds ratios are listed in the Supplementary Tables S6–S9.

## 4 Discussion

We conducted a systematic review of RCTs in which docetaxel-based systemic triplet therapy was administered to patients with mHSPC. Four systemic triplet therapies were identified in eligible trials, and we performed NMA to indirectly compare the efficacy and safety of these therapies. Several systematic reviews and NMAs of mHSPC have been published. However, most studies have only focused on and compared ADT plus docetaxel or ARTA, namely doublet therapy, or did not include recently published data (Thomas et al., 2021; Wang et al., 2021; Mori et al., 2022). In two recent studies, as a class of treatment strategy, the systemic triplet therapy for mHSPC has been evaluated and compared with doublet therapy, but the efficacy and safety of different triplet therapy has not been compared. In our study, we used ADT plus docetaxel combined with one ARTA, systemic triplet therapy, and included two recent RCTs (Fizazi et al., 2022; Smith et al., 2022). To our knowledge, this is the first study to indirectly compare different systemic triplet therapy for mHSPC using NMA. The findings of this study may provide valuable insights for clinical use.

In our NMA, ADT plus docetaxel combined with ARTA appeared to be more effective than ADT plus docetaxel in patients with mHSPC. The effectiveness of this chemohormonal triplet therapy may stem from the synergistic and complementary effects of the three agents. ADT is the treatment that inhibits tumor growth by lowering the levels of androgens made in testicles through medication or surgery. It is the cornerstone of hormonal therapy for PCa. ARTA, include the inhibitor of androgen synthesis (abiraterone) and antiandrogen (enzalutamide, apalutamide, and darolutamide) could suppress other sources of androgen to achieve maximum suppression of the androgen axis, and further improve the anti-tumor effect (Crawford et al., 2018). Docetaxel, a semisynthetic taxane, has exhibited significant antitumor activity through inhibiting microtubular depolymerization and attenuating the effects of bcl-2 and bcl-xL gene expression. Then the taxane-induced microtubule stabilization arrests cells in the G2M phase of the cell cycle and induces bcl-2 phosphorylation, promoting a cascade of events that ultimately leads to apoptotic cell death (Pienta, 2001). In addition, docetaxel could also affect androgen receptor (AR) signaling by inhibiting AR nuclear translocation and target gene transcription (Darshan et al., 2011). Thus, docetaxel could target castration-resistant prostate cancer cells *via* both AR-independent and AR-dependent activity synergistically and complementarily when combined with ADT. CHAARTED trial showed that six cycles of docetaxel at the beginning of ADT for mHSPC resulted in significantly longer OS than that with ADT alone (Sweeney et al., 2015). Therefore, when added with ARTA, which is the more potent suppressor for AR signaling, the triplet therapy may lead to better efficacy than ADT plus docetaxel through synergistic and complementary mechanisms.

Although systemic triplet therapy brought more benefits than doublet therapy, the efficacy of individual triplet therapy was various in NMA. The timing of docetaxel initiation and duration of therapy varied widely across these trials may affect effectiveness. In the ARCHES and TITAN trials, docetaxel was completed before enzalutamide or apalutamide administration, whereas in the ENZAMET trial, prior or concomitant docetaxel administration was acceptable. Furthermore, some of these patients did not complete the standard six cycles of docetaxel chemotherapy. In contrast, in the PEACE-1 and ARASENS trials, all patients completed the six cycles of docetaxel chemotherapy concomitantly. This may result in various antitumor effects based on the synergistic mechanisms of docetaxel and ARTA. A sensitivity analysis was performed by excluding ARCHES, TITAN and ENZAMET trials. The forest plot and SUCRA plot for OS and time to castration resistance in PEACE-1 and ARASENS trials were generated, revealing the same results for HRs and treatment ranking probabilities (Supplementary Figure S5). This suggested that the timing of docetaxel initiation and duration may not affect the outcomes of our NMA.

This also reminds us of the importance of timing and sequencing of chemohormonal therapy for prostate cancer. Before the introduction of systemic triplet therapy, while both ADT plus docetaxel and ADT plus other ARTAs have shown their effectiveness for mHSPC, the optimal timing and sequence to employing each therapy has not been well-defined. Preclinical evidence has been demonstrated that simultaneous chemohormonal therapy were more effective than sequential treatment (Eigl et al., 2005). In our NMA, better efficacy was also shown in trials with adequate and simultaneous docetaxel-combined arms than ADT plus docetaxel alone. This finding was consistent with a recent meta-analysis (Fallara et al., 2022). However, in that study and another network meta-analysis, it was suggested that compared with doublet therapy of ADT plus ARTA, no OS and PFS benefit was found for patients with mHSPC treated with triplet therapy (Fallara et al., 2022; Roy et al., 2022). Therefore, among current available treatment strategies for mHSPC, ADT plus docetaxel alone is the last choice compared with other therapies. Whether the systemic triplet therapy of ADT, ARTA, and docetaxel or the doublet of ADT plus ARTA is the first-line treatment strategy for mHSPC remains controversial.

The safety of systemic triplet therapy is comparable to that of ADT plus docetaxel. When evaluating the efficacy of systemic triplet therapy, safety is also a concern. The comparator therapy was ADT plus docetaxel in this study; hence, we were concerned with two questions: 1) whether the combination of agents in the triplet therapy increases the toxicity of the individual agents and; 2) whether there are any new AEs from the triplet therapy. Due to comparable outcomes, we focused on darolutamide and abiraterone triplet therapy. Docetaxel is associated with a high risk of fatigue, neuropathy, and myelosuppression, including neutropenia, anemia, and thrombocytopenia (Mori et al., 2022). Compared with ADT plus docetaxel in the included trials, the darolutamide triplet therapy was associated with a higher risk of hypertension. In comparison, the abiraterone triplet therapy was associated with a higher risk of hypertension and hepatotoxicity. However, other docetaxel-related AEs did not increase significantly compared with the comparator in both therapies. The higher risk of hypertension and hepatotoxicity in the abiraterone triplet therapy may be related to the addition of abiraterone (Fizazi et al., 2017). Intriguingly, the darolutamide triplet therapy also increased hypertension incidence, which was not observed in previous darolutamide or docetaxel RCTs on mHSPC (Sweeney et al., 2015; Fizazi et al., 2019). In our NMA, the risk of hypertension in both darolutamide and abiraterone triplet therapies was comparable and significantly higher than that in doublet ADT plus docetaxel. Overall, any observed AE in these two triplet therapies was comparable to that in ADT plus docetaxel. However, due to the low risk of grade ≥3 AE and hepatotoxicity, darolutamide triplet therapy has a slight advantage.

The results of this NMA must be interpreted with caution. Although there was no significant difference in OS improvement between any two of the five triplet therapies, it was unexpected that enzalutamide and apalutamide triplet therapies did not show superiority compared to ADT plus docetaxel. The data for these two triplet therapies came from subgroups of prior or early docetaxel therapy in the ARCHES and TITAN trials. Interestingly, all of these trials obtained better OS when analyzed in the overall population; however, the efficacy of the prior or early docetaxel subgroup was not as good as that of the overall population or even failed to improve OS. The contradictory outcome may be due to study design. These trials were neither designed nor powered to analyze the effects of triplet therapy. Limited sample size in the subgroup may lead to false-negative or false-positive results (Brookes et al., 2001). Another controversial example is the PEACE-1 trial with a 2 × 2 factorial design. The patients in this trial were randomly assigned (1:1:1:1) to arm A (ADT plus docetaxel), arm B (ADT plus docetaxel plus abiraterone), arm C (ADT plus docetaxel plus radiotherapy), or arm D (ADT plus docetaxel plus radiotherapy plus abiraterone). Overall survival in arm B vs. A and arm D vs. C did not improve (HR 0.73, 95% CI: 0.52–1.03; HR 0.76, 95% CI: 0.54–1.08), showing that the abiraterone triplet was not superior to ADT plus docetaxel in the respective comparison. Based on the assumption that there was no significant interaction between radiotherapy and abiraterone (*p* = 0.85), arms B and D, arms A and C were pooled respectively for 2 × 2 analysis for abiraterone efficacy. Here, arms B and D were equivalent to two subgroups of ADT plus docetaxel plus abiraterone triplet therapy. If we had insisted on analyzing the data from arms B and A (“pure” triplet and doublet without radiotherapy) in this trial, there would have been a high risk of false-negative results. Therefore, we used pooled data for analysis in our study, as in the previous study (Fizazi et al., 2022).

This study has some limitations. First, due to the limitation of published data, some triplet therapy data in the subgroups were not obtained for analysis. Second, the subgroup analyses, like any comparison of non-randomized treatment groups, can be potentially biased due to unobserved or unmeasured confounding factors. As discussed above, inappropriate subgroup analysis may be misleading and yield false-negative or false-positive results. In addition, different trial designs may have an impact on treatment outcomes. For example, the stage of metastasis at the initial diagnosis varies. In the PEACE-1 trial, all included patients had *de novo* mHSPC, while in the ARCHES, ENZAMET, TITAN, and ARASENS trials, the metastatic rates were 66.7%, 72%, 86.3%, and 86%, respectively when patients were diagnosed. Metachronous metastatic status influences efficacy outcomes (Sweeney et al., 2021). Other factors may include the proportion of the high or low volume of disease and subsequent agents. In PEACE-1 trial, the abiraterone triplet therapy demonstrated better benefits on OS and rPFS for patients with high volume than with low volume. However, in ENZAMET trial, patients with low volume benefited more from enzalutamide triplet therapy (Supplementary Table S10). Due to limited published data, it was impossible to conduct a meta-analysis after adjustment or subgroup analysis in NMA. Finally, although NMA has been widely used and validated to indirectly compare outcomes from RCTs, the results would not be a substitute for direct head-to-head comparisons. However, due to the lack of direct comparisons for systemic triplet therapy, this is currently the best comparative evidence we could provide between these treatments, and more well-designed trials are expected.

Due to these limitations, more well-designed head-to-head clinical trials are required to evaluate the efficacy and safety of current triplet therapies for mHSPC. Also, additional triplet therapy combinations need to be studied. The combination of ADT plus abiraterone plus apalutamide extended rPFS for metastatic castration-resistant prostate cancer but did not improve OS (Saad et al., 2021), and this triplet therapy has not been evaluated for mHSPC. Some trials with the combination of PARP (Poly [ADP-ribose] polymerase) or PI3K (phosphatidylinositol 3-kinase)/AKT(protein kinase B) inhibitors are being studied in mHSPC. For instance, TALAPRO-3 trial (ADT plus enzalutamide plus talazoparib) and CAPItello-281 trial (ADT plus abiraterone plus capivasertib) (Fizazi et al., 2021; Agarwal et al., 2022). Moreover, the recent NMA study have shown that the triplet therapy did not confer a statistically significant OS benefit over ADT plus ARTA, suggesting that docetaxel, when given as part of triplet therapy, has limited independent OS benefit (Roy et al., 2022). Previous NMA studies have also shown that ADT plus some novel ARTA is inherently superior to ADT plus docetaxel for mHSPC treatment (Wang et al., 2021; Mori et al., 2022). Thus, the extent to which docetaxel plays a role in systemic triplet therapy compared with ADT plus ARTA is questionable. More head-to-head clinical trial comparing triplet therapy with ADT plus novel ARTA and direct comparison are needed.

## 5 Conclusion

In this systematic review and NMA, we indirectly compared the efficacy and safety of docetaxel-based systemic triplet therapies in patients with mHSPC. We observed that abiraterone triplet therapy improved OS and rPFS than standard ADT plus docetaxel doublet therapy. Darolutamide and enzalutamide triplet therapies improved OS and rPFS, respectively. Although more agents were used in triplet therapies, the adverse events did not increase significantly. These findings may help inform and guide clinicians in deciding the most individualized treatment for their patients. With more ongoing trials, more combinations of systemic triplet therapy will be proposed or become the standard of care for mHSPC in the future.

## Data Availability

The original contributions presented in the study are included in the article/Supplementary Material, further inquiries can be directed to the corresponding author.
