# Supplementary material for: Systemic triplet therapy for metastatic hormone-sensitive prostate cancer: A systematic review and network meta-analysis
Source: Front Pharmacol. 2022 Oct 6;13:955925. doi: 10.3389/fphar.2022.955925 (PMC9582339; doi:10.3389/fphar.2022.955925)
Supplement: Supplementary file 2 [file DataSheet1.PDF]

Supplementary Table 1 Search strategies in this systematic review

Supplementary Table 1.1 Search strategy in Pubmed

#1	"Prostatic Neoplasms"[Mesh]
#2	(Prostate Neoplasms[Title/Abstract]) OR (Neoplasms, Prostate[Title/Abstract]) OR (Neoplasm, Prostate[Title/Abstract]) OR (Prostate Neoplasm[Title/Abstract]) OR (Neoplasms, Prostatic[Title/Abstract]) OR (Neoplasm, Prostatic[Title/Abstract]) OR (Prostatic Neoplasm[Title/Abstract]) OR (Prostate Cancer[Title/Abstract]) OR (Cancer, Prostate[Title/Abstract]) OR (Cancers, Prostate[Title/Abstract]) OR (Prostate Cancers[Title/Abstract]) OR (Cancer of the Prostate[Title/Abstract]) OR (Prostatic Cancer[Title/Abstract]) OR (Cancer, Prostatic[Title/Abstract]) OR (Cancers, Prostatic[Title/Abstract]) OR (Prostatic Cancers[Title/Abstract]) OR (Cancer of Prostate[Title/Abstract])
#3	#1 OR #2
#4	"Neoplasm Metastasis"[Mesh]
#5	(Neoplasm Metastases[Title/Abstract]) OR (Metastases, Neoplasm[Title/Abstract]) OR (Metastasis, Neoplasm[Title/Abstract]) OR (Metastase[Title/Abstract]) OR (Metastases[Title/Abstract]) OR (Metastasis[Title/Abstract]) OR (metastatic[Title/Abstract])
#6	#4 OR #5
#7	"Docetaxel"[Mesh]
#8	(Docetaxel Trihydrate[Title/Abstract]) OR (Docetaxol[Title/Abstract]) OR (Docetaxel Hydrate[Title/Abstract]) OR (Taxoltere Metro[Title/Abstract]) OR (RP 56976[Title/Abstract]) OR (RP-56976[Title/Abstract]) OR (RP56976[Title/Abstract]) OR (Taxotere[Title/Abstract]) OR (Docetaxel Anhydrous[Title/Abstract]) OR (N-Debenzoyl-N-tert-butoxycarbonyl-10-deacetyltaxol[Title/Abstract]) OR (N Debenzoyl N tert butoxycarbonyl 10 deacetyltaxol[Title/Abstract]) OR (NSC 628503[Title/Abstract])
#9	#7 OR #8
#10	#3 AND #6 AND #9
#11	randomized controlled trial[Publication Type] OR randomized[Title/Abstract] OR placebo[Title/Abstract]
#12	#10 AND #11

Supplementary Table 1.2 Search strategy in Embase

#1	'prostate cancer'/exp
#2	'prostate neoplasms':ab,ti,kw OR 'neoplasms, prostate':ab,ti,kw OR 'neoplasm, prostate':ab,ti,kw OR 'prostate neoplasm':ab,ti,kw OR 'neoplasms, prostatic':ab,ti,kw OR 'neoplasm, prostatic':ab,ti,kw OR 'prostatic neoplasm':ab,ti,kw OR 'prostate cancer':ab,ti,kw OR 'cancer, prostate':ab,ti,kw OR 'cancers, prostate':ab,ti,kw OR 'prostate cancers':ab,ti,kw OR 'cancer of the prostate':ab,ti,kw OR 'prostatic cancer':ab,ti,kw OR 'cancer,

	prostatic':ab,ti,kw OR 'cancers, prostatic':ab,ti,kw OR 'prostatic cancers':ab,ti,kw OR 'cancer of prostate':ab,ti,kw
#3	#1 OR #2
#4	'metastasis'/exp
#5	('neoplasm metastases':ab,ti,kw OR 'metastases, neoplasm':ab,ti,kw OR 'metastasis, neoplasm':ab,ti,kw OR 'metastase':ab,ti,kw OR 'metastases':ab,ti,kw OR 'metastasis':ab,ti,kw) AND 'metastatic':ti,ab,kw
#6	#4 OR #5
#7	'docetaxel'/exp
#8	'docetaxel trihydrate':ab,ti,kw OR 'docetaxol':ab,ti,kw OR 'docetaxel hydrate':ab,ti,kw OR 'taxoltere metro':ab,ti,kw OR 'rp 56976':ab,ti,kw OR 'rp-56976':ab,ti,kw OR 'rp56976':ab,ti,kw OR 'taxotere':ab,ti,kw OR 'docetaxel anhydrous':ab,ti,kw OR 'n-debenzoyl-n-tert-butoxycarbonyl-10-deacetyltaxol':ab,ti,kw OR 'n debenzoyl n tert butoxycarbonyl 10 deacetyltaxol':ab,ti,kw OR 'nsc 628503':ab,ti,kw
#9	#7 OR #8
#10	#3 AND #6 AND #9
#11	'randomized controlled trial'/exp
#12	#10 AND #11

Supplementary Table 1.3 Search strategy in Cochrane CENTRAL

#1	MeSH descriptor: [Prostatic Neoplasms] explode all trees
#2	(Prostate Neoplasms):ti,ab,kw OR (Neoplasms, Prostate):ti,ab,kw OR (Neoplasm, Prostate):ti,ab,kw OR (Prostate Neoplasm):ti,ab,kw OR (Neoplasms, Prostatic):ti,ab,kw OR (Neoplasm, Prostatic):ti,ab,kw OR (Prostatic Neoplasm):ti,ab,kw OR (Prostate Cancer):ti,ab,kw OR (Cancer, Prostate):ti,ab,kw OR (Cancers, Prostate):ti,ab,kw OR (Prostate Cancers):ti,ab,kw OR (Cancer of the Prostate):ti,ab,kw OR (Prostatic Cancer):ti,ab,kw OR (Cancer, Prostatic):ti,ab,kw OR (Cancers, Prostatic):ti,ab,kw OR (Prostatic Cancers):ti,ab,kw OR (Cancer of Prostate):ti,ab,kw
#3	(#1 OR #2)
#4	MeSH descriptor: [Neoplasms Metastasis] explode all trees
#5	(Neoplasm Metastases):ti,ab,kw OR (Metastases, Neoplasm):ti,ab,kw OR (Metastasis, Neoplasm):ti,ab,kw OR (Metastase):ti,ab,kw OR (Metastases):ti,ab,kw OR (Metastasis):ti,ab,kw OR (metastatic):ti,ab,kw
#6	(#4 OR #5)
#7	MeSH descriptor: [Docetaxel] explode all trees
#8	(Docetaxel Trihydrate):ti,ab,kw OR (Docetaxol):ti,ab,kw OR (Docetaxel Hydrate):ti,ab,kw OR (Taxoltere Metro):ti,ab,kw OR (RP 56976):ti,ab,kw OR (RP-56976):ti,ab,kw OR (RP56976):ti,ab,kw OR (Taxotere):ti,ab,kw OR (Docetaxel Anhydrous):ti,ab,kw OR (N Debenzoyl N tert butoxycarbonyl 10 deacetyltaxol):ti,ab,kw OR (NSC 628503):ab,ti,kw
#9	(#7 OR #8)
#10	(#3 AND #6 AND #9)

Supplementary Table 2 Outcomes Assessed in Included Trials in Network Meta-Analysis

Trial ID	Outcome	Outcome definition
ARCHES	Overall survival	Time from randomization to death from any cause
	Radiographic progression-free survival	Time from randomization to the first objective evidence of radiographic disease progression, or death, whichever occurred first. Radiographic disease progression is defined by RECTST version 1.1 for soft tissue disease or the appearance of 2 or more new lesions on bone scan
	Time to PSA progression	Time from randomization to a >=25% increase and an absolute increase of >=2 ng/ml above the nadiar, which is confirmed by a second consecutive value at least 3 weeks later
	Time to initiation of new antineoplastic therapy	Time from randomization to the initiation of antineoplastic therapy (including cytotoxic and hormonal therapy) subsequent to the study treatments
	Time to castration resistance	Time from randomization to the first castration-resistant event (radiographic disease progression, PSA progression or symptomatic skeletal event with castration levels of testosterone, whichever occurs first.
	Time to first symptomatic skeletal event	The time from randomization to the occurrence of the first symptomatic skeletal event, defined as radiation or surgery to bone, clinically apparent pathologic bone fracture, or spinal cord compression
ENZAMET	Overall survival	The interval from randomization to death from any cause
	Clinical progression-free survival	The earliest sign of radiographic progression according to the criteria of the PCWG 2 for bone lesions and RECIST, version 1.1, for soft-tissue lesions; the development of symptoms attributable to cancer progression; or the initiation of another anticancer treatment for prostate cancer.
	PSA progression-free survival	The interval from randomization to the earliest event of PSA progression according to the criteria of PCWG2, clinical progression, death from any cause

Trial ID	Outcome	Outcome definition
TITAN	Overall survival	Time from randomization to the date of death from any cause
	Radiographic progression-free survival	Time from randomization to first imaging-based documentation of progressive disease or death, whichever occurred first. Radiographic progressive disease: progression of soft-tissue lesions according to modified RECIST, version 1.1, or new bone lesions according to PCWG 2.
PEACE-1	Overall survival	The time between randomisation and death from any cause.
	Radiographic progression-free survival	The time between randomisation and the occurrence of radiographical progression or death from any cause.Radiographical progression of soft-tissue lesions was evaluated by either CT or MRI, on the basis of RECIST version 1.1. Progression of bone lesions was assessed by bone scan according to the adapted version of Prostate Cancer Working Group 2 criteria,with no secondary bone scan required to confirm progression.
	CRPC-free survival	The time between randomisation and CRPC or death from any cause. CRPC was defined as either radiographical progression or a confirmed PSA rise (based on three indepen dent measurements: A, B, and C, with A<B<C, and C≥0·50 ng/mL), with a serum testosterone within castrated range (<0·50 ng/mL).
ARASENS	Overall survival	The time (in days) from date of randomization until death from any cause.
	Time to castration-resistant prostate cancer	The time to PSA progression with serum testosterone being at castrate level <0.50 ng/mL, or the time to progression by soft tissue lesions or time to progression by bone lesions, whatever comes first.
	Time to first symptomatic skeletal event	The time from randomization to the first occurrence of SSE. An SSE is defined as EBRT to relieve skeletal symptoms, or new symptomatic pathologic bone fracture, or occurrence of spinal cord compression or tumor-related orthopedic surgical intervention, whichever comes first.
	Time to initiation of subsequent systemic antineoplastic therapy	The time from randomization to initiation of first subsequent antineoplastic therapy for prostate cancer.

Supplementary Table 3 Results of Efficacy outcomes of Included Trials in Network Meta-Analysis

Trial ID	Experimental (added to ADT and Docetaxel)	Comparator (added to ADT and Docetaxel)	OS,HR(95%CI)	rPFS, HR (95%CI)	Time to CRPC, HR (95%CI)	Time to PSA progression, HR (95%CI)	Time to first SSE, HR(95%CI)	Time to new antineoplastic therapy, HR(95%CI)
			Experimental vs comparator	Experimental vs comparator	Experimental vs comparator	Experimental vs comparator	Experimental vs comparator	Experimental vs comparator
**ARCHES**	Enzalutamide	Placebo	0.74(0.46-1.2)	0.52(0.30-0.89)	0.41(0.25-0.67)	0.22(0.11-0.45)	0.85(0.39-1.87)	0.40(0.21-0.77)
**ENZAMET**	Enzalutamide	SNA	0.9(0.62-1.31)	0.48(0.37-0.62)[a]	Not reported	0.46(0.36-0.60)[c]	Not reported	Not reported
**TITAN**	Apalutamide	Placebo	1.12(0.59-2.12)	0.47(0.22-1.01)	Not reported	Not reported	Not reported	Not reported
**PEACE-1**	Abiraterone+ RT(+/-)	RT(+/-)	0.75(0.59-0.95)[d]	0.50(0.39-0.61)[e]	0.38(0.31-0.47)[b]	Not reported	Not reported	Not reported
**ARASENS**	Darolutamide	Placebo	0.68(0.57-0.80)	Not reported	0.36(0.30-0.42)	Not reported	0.71(0.54-0.94)	0.39(0.33-0.46)

RT: Radiotherapy; SNA: standard nonsteroidal antiandrogen (bicalutamide, nilutamide or flutamide); SSE: Symptomatic skeletal event

a. Clinical Progression-free survival

b. CRPC-free survival

c. PSA progression-free survival

d. For comparison, the value has been transferred from 95.1%CI to 95%CI

e. For comparison, the value has been transferred from 99.9%CI to 95%CI

Supplementary Table 4 Detailed Assessment of Risk of Bias in Included Trials in Network Meta-Analysis

Type of bias	Domains in the tool	ARCHES	ENZAMET	TITAN	PEACE-1	ARASENS
**Selection bias**	**Sequence generation**	"Randomization was performed centrally by tele-phone with the use of a computerized algorithm" **comment:low risk**	"In this open-label, randomized, phase 3 trial,..." **comment:unclear**	"The investigators, patients, trial-site personnel, and sponsor trial team were unaware of the randomization codes until trial completion" **Comment:low risk**	"This randomisation process was performed via the Tenalea autonomous software," **Comment:low risk**	"In this international, randomized, double-blind, placebo-controlled trial..." **Comment:unclear**
	**Allocation concealment**	"Randomization was performed centrally by tele-phone with the use of a computerized algorithm" **Comment:low risk**	"The central randomization system implement-ed minimization with a random component" **Comment:low risk**	"The investigators, patients, trial-site personnel, and sponsor trial team were unaware of the randomization codes until trial completion" **Comment:low risk**	"Randomisation was done using a minimisation algorithm," **Comment:low risk**	"Participants Randomized by Center" **Comment:low risk**
**Performance bias**	**Blinding of participants and personnel**	Masking: Quadruple (Participant, Care Provider, Investigator, Outcomes Assessor) **Comment:low risk**	"this multinational, openlabel, randomized, phase 3 trial..." **Comment:high risk**	"The investigators, patients, trial-site personnel, and sponsor trial team were unaware of the randomization codes until trial completion" **Comment:low risk**	"an open-label, randomised, active-controlled, phase 3 study" **Comment:high risk**	Masking: Quadruple (Participant, Care Provider, Investigator, Outcomes Assessor) **Comment:low risk**

**Detection bias**	**Blinding of outcome assessment**	"processed data that were released by the independent data monitoring committee and trial steering committee were available to all the coauthors." **Comment:low risk**	"An independent data and safety monitoring committee reviewed the progress and results of the trial." **Comment:low risk**	"An independent data-monitoring committee was commissioned by the sponsor to monitor safety and efficacy before unblinding and to make recommendations regarding trial conduct." **Comment:low risk**	"Steering and Independent Data Monitoring Committees roles and members are described in the appendix" **Comment:low risk**	"An independent data and safety monitoring board reviewed unblinded safety and efficacy data throughout the trial." **Comment:low risk**
**Attrition bias**	**Incomplete outcome data**	The number of missing persons and reasons were similar between groups. **Comment:low risk**	Missing data are not sufficient to affect effect values. **Comment:low risk**	The number of missing persons and reasons were similar between groups. **Comment:low risk**	Missing data are not sufficient to affect effect values. **Comment:low risk**	Missing data are not sufficient to affect effect values. **Comment:low risk**
**Reporting bias**	**Selective outcome reporting**	The study report is the same as the study protocol registered with ClinicalTrial .(NCT02677896) **Comment:low risk**	The study report is the same as the study protocol registered with ClinicalTrial. （NCT02446405） **Comment:low risk**	The study report is the same as the study protocol registered with ClinicalTrial. (NCT02489318) **Comment:low risk**	The study report is the same as the study protocol registered with ClinicalTrial. (NCT01957436) **Comment:low risk**	The study report is the same as the study protocol registered with ClinicalTrial. （NCT02799602） **Comment:low risk**
**Bias from other sources**	**Baseline for the intervention and control groups**	The intervention and control groups were similar at baseline. **Comment:low risk**	The intervention and control groups were similar at baseline. **Comment:low risk**	The intervention and control groups were similar at baseline. **Comment:low risk**	The intervention and control groups were similar at baseline. **Comment:low risk**	The intervention and control groups were similar at baseline. **Comment:low risk**

	**Source of funding**	“Further funding for the platform was provided by Astellas Pharma, Clovis Oncology, Novartis, Pfizer, and Sanofi Aventis” **Comment:unclear**	“representatives of the company reviewed drafts of the protocol and trial report but were not otherwise involved in any aspects of the trial design, data accrual, data analysis, or manuscript preparation” **Comment:low risk**	“Funded by Janssen Research and Development” **Comment:unclear**	“The funders of the study had no role in study design, data collection, data analysis, data interpretation, or writing of the report.” **Comment:low risk**	“Funded by Bayer and Orion Pharma” **Comment:unclear**

Supplementary Table 5 Certainty of the evidence for each outcomes based on the GRADE approach

Outcomes	Certainty of the evidence (GRADE)
Overall survival	⊕⊕⊕⊖<br>Moderate[1]
Radiographic progression-free survival	⊕⊕⊕⊖<br>Moderate[1]
Time to castration resistance	⊕⊕⊕⊖<br>Moderate[1]
Time to PSA progression	⊕⊕⊕⊖<br>Moderate[1]
Time to first symptomatic skeletal event	⊕⊕⊕⊕<br>High
Time to new antineoplastic therapy	⊕⊕⊕⊕<br>High
Adverse events (any grade)	⊕⊕⊖⊖<br>Low[1,2]
Adverse events (≥ grade 3)	⊕⊕⊕⊖<br>Moderate[1]
Hypertension	⊕⊕⊕⊖<br>Moderate[1]
Neutropenia	⊕⊕⊕⊖<br>Moderate[1]
Febrile neutropenia	⊕⊕⊕⊖<br>Moderate[1]

[1]Study limitation (risk of bias)
[2]Imprecision

1.Study limitation(risk of bias)

Author's adjustment	Individual studies	Studies
**Low risk of bias**	All 7 items were assessed as low risk of bias	
**Unclear**	One or more items were assessed as unclear	ARCHES, TITAN, ARASENS
**High risk of bias**	One or more items were assessed as high risk of bias	ENZAMET, PEACE-1

SO this aspect affect the assessment of the level of evidence.

2.Indirectness:The populations, interventions and outcome indicators in the text are equivalent to the researchers' concerns in reality. So this aspect does not affect the assessment of the level of evidence.

3.Inconsistency: Firstly, Similar baseline data between different studies. Secondly,there was no merging between identical comparisons. SO this aspect does not affect the assessment of the level of evidence.

4.Imprecision: The interval of results is too wide or there are studies with small study samples. SO this aspect affect the assessment of the level of evidence.

5.Publication bias: The study report is the same as the study protocol registered. SO this aspect does not affect the assessment of the level of evidence.

GRADE Working Group grades of evidence
High certainty: We are very confident that the true effect lies close to that of the estimate of the effect
Moderate certainty: We are moderately confident in the effect estimate; the true effect is likely to be close to the estimate of the effect, but there is a possibility that it is substantially different
Low certainty: Our confidence in the effect estimate is limited; the true effect may be substantially different from the estimate of the effect
Very low certainty: We have very little confidence in the effect estimate; the true effect is likely to be substantially different from the estimate of effect

Supplementary Table 6 Adverse Events in ARASENS Trial

Selected adverse events n (%)	Darolutamide+ADT +Docetaxel (652)	Placebo +ADT +Docetaxel (650)	OR(95%CI)
**Any adverse event**	649 (99.5)	643 (98.9)	2.36(0.61-9.15)
**Grade≥3 AE**	458 (70.2)	439 (67.5)	1.13(0.90-1.44)
**Selected grade 3 or 4 AE‡**			
Neutropenia*	220 (33.7)	222 (34.2)	0.98(0.78-1.23)
Febrile neutropenia	51 (7.8)	48 (7.4)	1.06(0.71-1.60)
Hypertension	42 (6.4)	21 (3.2)	2.06(1.21-3.52)
Anemia	31 (4.8)	33 (5.1)	0.93(0.56-1.54)
Pneumonia	21 (3.2)	20 (3.1)	1.05(0.56-1.95)
Hyperglycemia	18 (2.8)	24 (3.7)	0.74(0.40-1.38)
Increased ALT level	18 (2.8)	11 (1.7)	1.65(0.77-3.52)
Increased AST level	17 (2.6)	7 (1.1)	2.46(1.01-5.97)
Increased weight	14 (2.1)	8 (1.2)	1.76(0.73-4.23)
Urinary tract infection	13 (2.0)	12 (1.8)	1.08(0.49-2.39)

*The neutropenia category includes the preferred terms of leukopenia, neutropenia, decreased neutrophil count, and decreased white-cell count.

‡listed are all grade 3 or 4 events that occurred in at least 2% of the patients and are reported in decreasing order of occurrence

ADT:androgen deprivation therapy. ALT denotes alanine aminotransferase, and AST aspartate aminotransferase. OR:odds ratio

Supplementary Table 7 Adverse Events in PEACE-1 Trial

Selected adverse events n (%)	Abiraterone+ADT +Docetaxel (RXT+/-)(347)	ADT+Docetaxel (RXT+/-)(350)	OR(95%CI)
**Any adverse event**	346 (100%)	349 (100%)	0.99(0.06-15.91)
**Grade≥3 AE**	217 (63%)	181 (52%)	1.56(1.15-2.11)
**Selected grade≥3 AE‡**			
Hypertension	76 (22%)	45 (13%)	1.90(1.27-2.85)
Neutropenia	34 (10%)	32 (9%)	1.08(0.65-1.79)
Hepatotoxicity	20 (6%)	2 (1%)	10.64(2.47-45.89)
Febrile neutropenia	18 (5%)	19 (5%)	0.95(0.60-1.85)
Gamma-glutamyl transferase increase	17 (5%)	14 (4%)	1.24(0.60-2.55)
Blood alkaline phosphatase increase	15 (4%)	12 (3%)	1.27(0.59-2.76)

The safety population includes patients who actually received the assigned treatment; ‡listed are all grade≥3 events that occurred in at least 5% of the patients and are reported in decreasing order of occurrence.

ADT:androgen deprivation therapy. OR:odds ratio

Supplementary Table 8

ARASENS: Adverse Events commonly associated with ADT or androgen receptor pathway inhibitor therapy

Selected adverse events n (%)	Darolutamide + ADT +Docetaxel (N = 652)	Placebo + ADT + Docetaxel (N = 650)	OR(95%CI)
Fatigue	216 (33.1)	214 (32.9)	1.01(0.80-1.27)
Vasodilatation and flushing	133 (20.4)	141 (21.7)	0.93(0.71-1.21)
Rash †	108 (16.6)	88 (13.5)	1.27(0.93-1.72)
Diabetes mellitus and hyperglycemia	99 (15.2)	93 (14.3)	1.07(0.79-1.46)
Hypertension	89 (13.7)	60 (9.2)	1.55(1.10-2.20)
Cardiac disorder	71 (10.9)	76 (11.7)	0.92(0.65-1.30)
Cardiac arrhythmia *	52 (8.0)	55 (8.5)	0.94(0.63-1.39)
Coronary artery disorder *	19 (2.9)	13 (2.0)	1.47(0.72-3.00)
Heart failure *	4 (0.6)	13 (2.0)	0.30(0.10-0.93)
Bone fracture‡	49 (7.5)	33 (5.1)	1.52(0.96-2.40)
Falls, including accident	43 (6.6)	30 (4.6)	1.46(0.90-2.36)
Mental-impairment disorder	23 (3.5)	15 (2.3)	1.55(0.80-2.99)
Weight decreased	22 (3.4)	35 (5.4)	0.61(0.36-1.06)
Depressed-mood disorder	21 (3.2)	24 (3.7)	0.87(0.48-1.58)
Breast disorders/gynecomastia	21 (3.2)	10 (1.5)	2.13(1.00-4.56)
Cerebral ischemia	8 (1.2)	8 (1.2)	1.00(0.37-2.67)
Seizure	4 (0.6)	1 (0.2)	4.01(0.45-35.94)

†This category combines the following MedDRA terms: rash, maculopapular rash, drug eruption, pruritic rash, erythematous rash, macular rash, papular rash, follicular rash, pustular rash, and vesicular rash.

* This category is a MedDRA High-Level Group Term.

‡ Excluding pathologic fractures. This category combines the following MedDRA terms: any fractures and dislocations, limb fractures and dislocations, pelvic fractures and dislocations, skull fractures, facial bone fractures and dislocations, spinal fractures and dislocations, and thoracic cage fractures and dislocations.

ADT=androgen deprivation therapy. MedDRA denotes Medical Dictionary for Regulatory Activities; OR:odds ratio

Supplementary Table 9 Adverse Events in ARCHES Trial

Selected adverse events n (%)	Enzalutamide+ADT+Docetaxel (103)	Placebo +ADT+ Docetaxe (102)	OR(95%Crl)
Overall	70 (68.0)	64 (62.7)	1.26(0.71-2.24)
Fatigue	34 (33.0)	30 (29.4)	1.18(0.65-2.14)
Hypertension	10 (9.7)	9 (8.8)	1.11(0.43-2.86)
Fall	6 (5.8)	4 (3.9)	1.52(0.41-5.54)
Cognitive/memory impairment	7 (6.8)	1 (1.0)	7.36(0.89-60.98)
Rash	5 (4.9)	2 (2.0)	2.55(0.48-13.46)

ARCHES : Treatment-emergent AEs of interest by prior docetaxel treatment; OR:odds ratio

Supplementary Table 10 Efficacy in subgroups of high and low volume in PEACE-1 and ENZAMET Trials

PEACE-1

	n(%)	OS,HR(95CrI)	rPFS,HR(95CrI)
**overall population**	710(100%)	0.75 (0.59–0.95)	0.50(0.39-0.61)
**high volume**			
ADT+DOC+ABI	224(32%)	0.72(0.55-0.95)	0.47(0.35-0.60)
ADT+DOC	232(33%)		
**low volume**			
ADT+DOC+ABI	131(18%)	0.83(0.50-1.39)	0.58(0.38-0.87)
ADT+DOC	123(17%)		

ENZAMET

	OS		cPFS	
	n(%)	HR(95CrI)	n(%)	HR(95CrI)
**overall population**	107(100%)	0.90(0.62-1.31)	237(100%)	0.48(0.37-0.62)
**high volume**				
ADT+DOC+ENZA	45(42%)	0.97(0.64-1.46)	75(32%)	0.51(0.38-0.69)
ADT+DOC+SNA	45(42%)		113(48%)	
**low volume**				
ADT+DOC+ENZA	7(6.5%)	0.65(0.25-1.71)	16(6.8%)	0.37(0.20-0.67)
ADT+DOC+SNA	10(9.3%)		33(14%)	

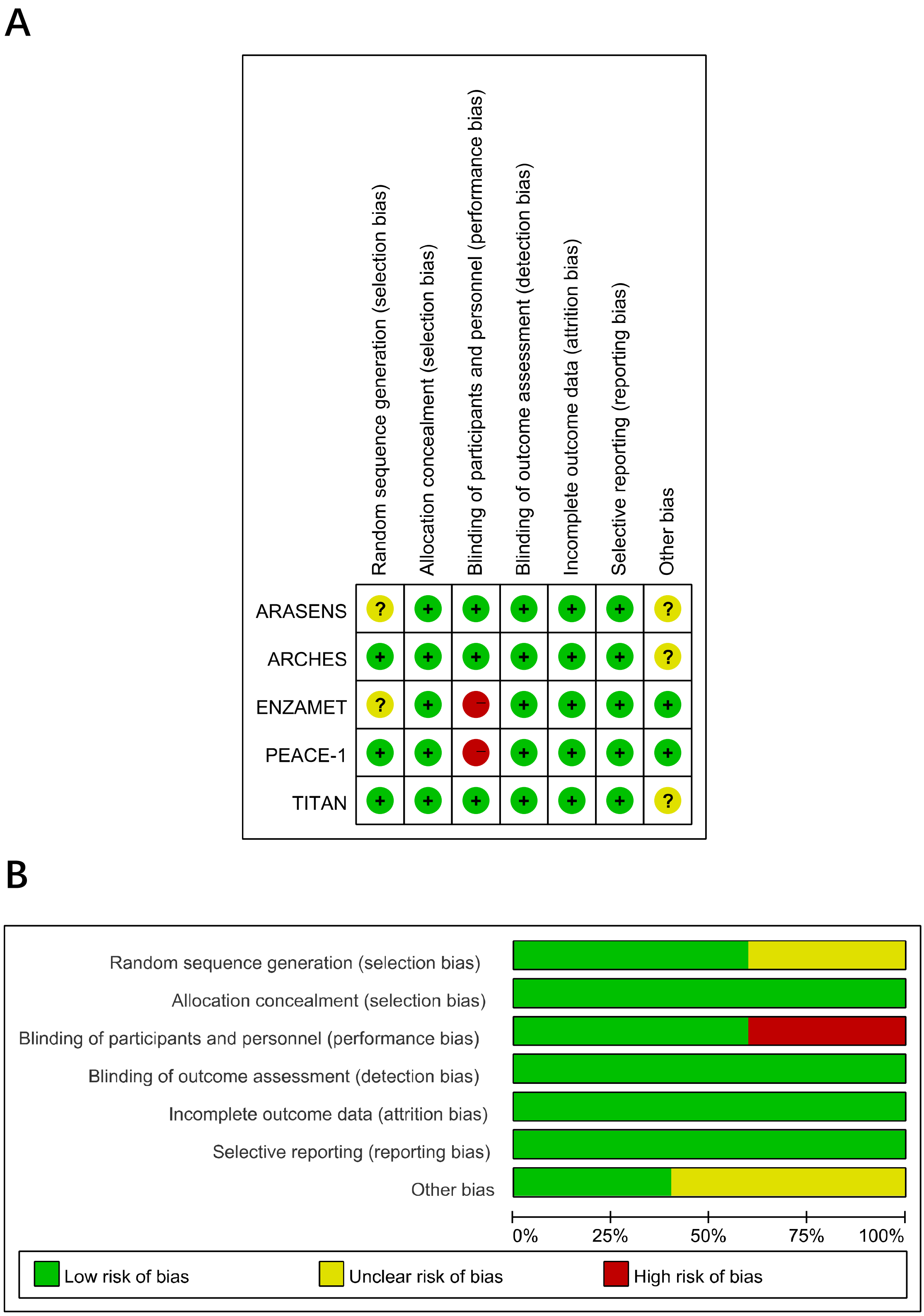

Supplementary Figure 1. Assessment of risk of bias of included trials. (A) Risk of bias summary for each RCT assessed according to the methods recommended by the Cochrane Collaboration. The green positive sign, low risk of bias; the red negative sign, high risk of bias; the yellow question mark, unclear risk of bias; (B) Risk of bias graph about each risk of bias item illustrated as percentage across all selected RCTs.

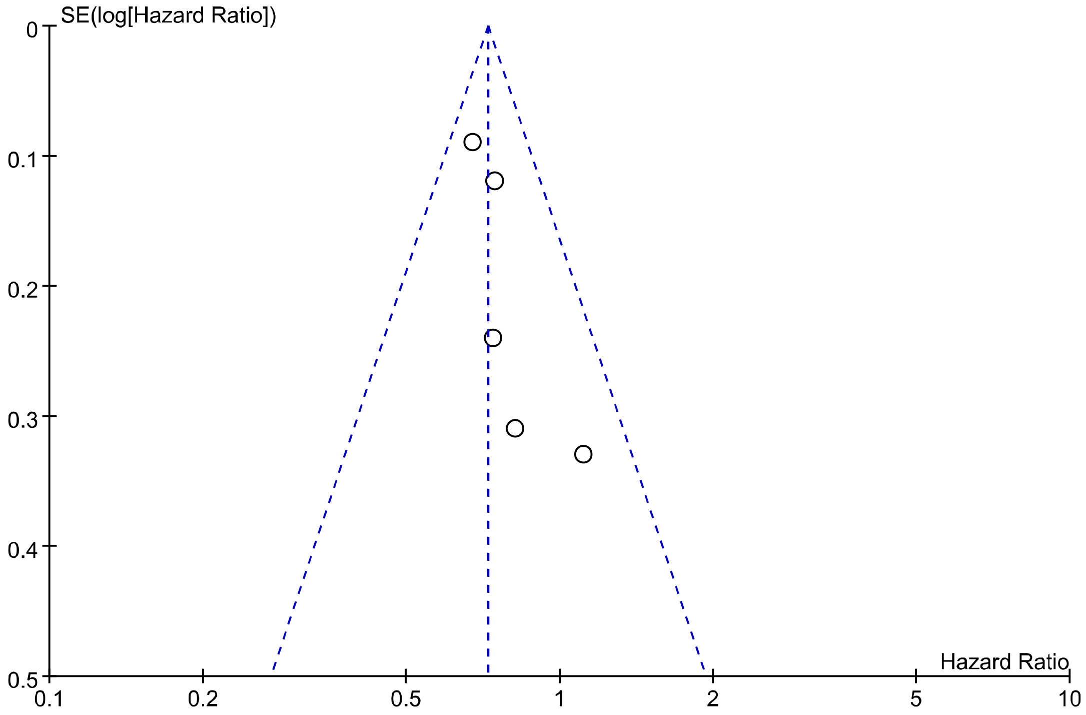

Supplementary Figure 2. Funnel plot showing symmetrical distribution of RCTs indicating absence of publication bias.

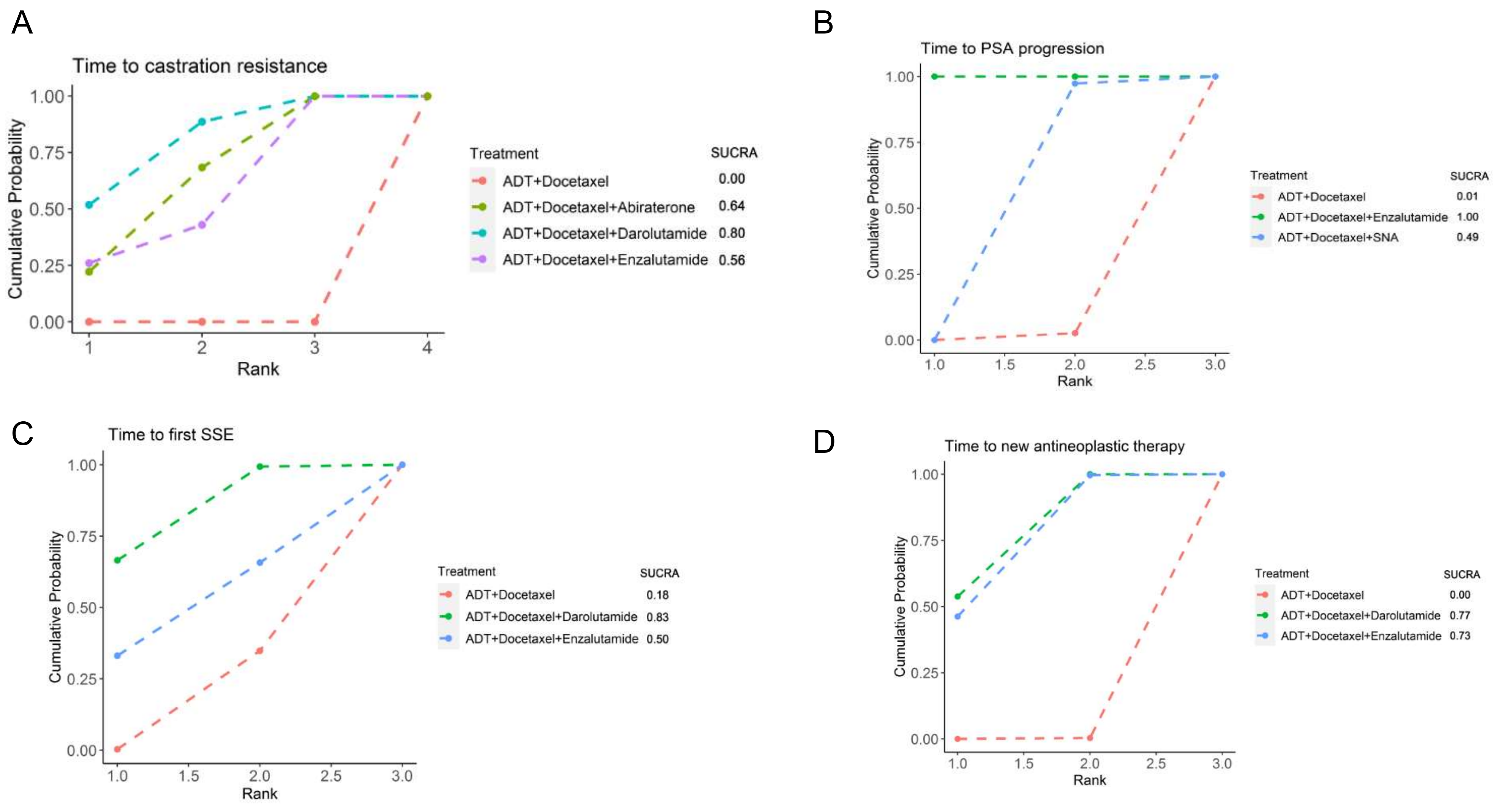

Supplementary Figure 3. SUCRA plot showing the treatment ranking of triplet therapy. (A) time to castration resistance; (B) time to PSA progression; (C) time to first SSE; (D) time to initiation of new antineoplastic therapy.

A. Time to CRPC

ADT+DOC	0.35 (0.30, 0.42)	0.41 (0.25, 0.67)	0.38 (0.31, 0.47)
2.82 (2.39, 3.32)	ADT+DOC+DARO	1.15 (0.68, 1.93)	1.08 (0.82, 1.41)
2.44 (1.50, 4.00)	0.87 (0.52, 1.47)	ADT+DOC+ENZA	0.93 (0.55, 1.60)
2.62 (2.12, 3.23)	0.93 (0.71, 1.22)	1.08 (0.63, 1.83)	ADT+DOC+ABI

B. Time to PSA progression

ADT+DOC	0.22 (0.11, 0.45)	0.48 (0.23, 1.01)
4.48 (2.24, 9.00)	ADT+DOC+ENZA	2.15 (1.66, 2.79)
2.08 (0.99, 4.44)	0.46 (0.36, 0.60)	ADT+DOC+SNA

C. Time to first SSE

ADT+DOC	0.71 (0.54, 0.94)	0.85 (0.39, 1.86)
1.40 (1.06, 1.84)	ADT+DOC+DARO	1.20 (0.52, 2.75)
1.17 (0.53, 2.57)	0.83 (0.36, 1.91)	ADT+DOC+ENZA

D. Time to initiation of new antineoplastic therapy

ADT+DOC	0.39 (0.33, 0.46)	0.40 (0.21, 0.77)
2.57 (2.18, 3.03)	ADT+DOC+DARO	1.03 (0.53, 2.01)
2.49 (1.30, 4.76)	0.97 (0.50, 1.90)	ADT+DOC+ENZA

Supplementary Figure 4. League table of network meta-analysis compare the secondary end points of systemic triplet therapy. Comparison is located at the intersection of the column-defining treatment and the row-defining treatment. The results are presented in HR with 95% CrI. HR>1 (red color) favors row-defining treatment, HR<1 (green color) favors column-defining treatment. Dark red or green color represent the results are statistically significant.

ADT: androgen deprivation therapy, SNA: Standard nonsteroidal antiandrogen, DOC: Docetaxel, ABI: Abiraterone, ENZA: Enzalutamide, DARO: Darolutamide, APA: Apalutamide

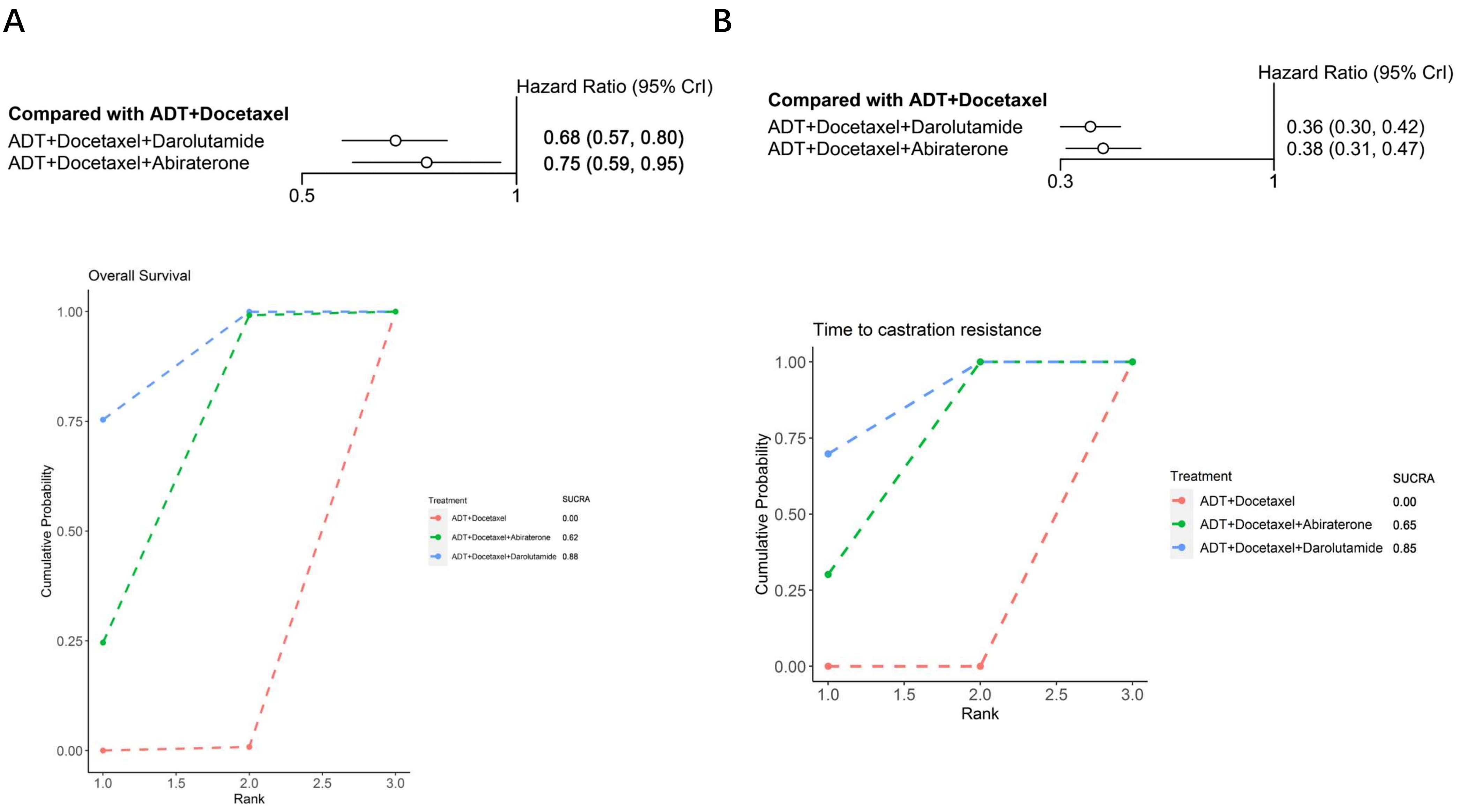

Supplementary Figure 5. Comparison of systemic triplet therapy after removing some trials in sensitivity analysis. (A) Upper: Forest plot representing HR on OS for darolutamide and abiraterone triplet therapy compared with ADT plus docetaxel. Down: SUCRA plot showing the treatment ranking of therapies. (B) Upper: Forest plot representing HR on time to castration resistance for darolutamide and abiraterone triplet therapy compared with ADT plus docetaxel. Down: SUCRA plot showing the treatment ranking of therapies.
